# Non-steroidal anti-inflammatory drugs and risk of gastric and oesophageal adenocarcinomas: results from a cohort study and a meta-analysis

**DOI:** 10.1038/sj.bjc.6604880

**Published:** 2009-01-20

**Authors:** C C Abnet, N D Freedman, F Kamangar, M F Leitzmann, A R Hollenbeck, A Schatzkin

**Affiliations:** 1 Nutritional Epidemiology Branch, Division of Cancer Epidemiology and Genetics, National Cancer Institute, Rockville, MD, USA; 2 AARP, Washington, DC, USA

**Keywords:** aspirin, NSAIDs, oesophageal cancer, gastric cancer, cohort, meta-analysis

## Abstract

Use of aspirin or other non-steroidal anti-inflammatory drugs (NSAIDs) may reduce the risk of gastric or oesophageal adenocarcinomas. We examined the association between self-reported use of aspirin or non-aspirin NSAIDs in the earlier 12 months and gastric non-cardia (*N*=182), gastric cardia (*N*=178), and oesophageal adenocarcinomas (*N*=228) in a prospective cohort (*N*=311 115) followed for 7 years. Hazard ratios (HRs) and 95% confidence intervals (CIs) come from Cox models adjusted for potential confounders. Use of any aspirin (HR, 95% CI: 0.64, 0.47–0.86) or other NSAIDs (0.68, 0.51–0.92) was associated with a significantly lower risk of gastric non-cardia adenocarcinoma. Neither aspirin (0.86, 0.61–1.20) nor other NSAIDs (0.91, 0.67–1.22) had a significant association with gastric cardia cancer. We found no significant association between using aspirin (1.00, 0.73–1.37) or other NSAIDs (0.90, 69–1.17) and oesophageal adenocarcinoma. We also performed a meta-analysis of the association between the use of NSAIDs and risk of gastric and oesophageal adenocarcinoma. In this analysis, aspirin use was inversely associated with both gastric and oesophageal adenocarcinomas, with summary odds ratios (95% CI) for non-cardia, cardia, and oesophageal adenocarcinomas of 0.64 (0.52–0.80), 0.82 (0.65–1.04), and 0.64 (0.52–0.79), respectively. The corresponding numbers for other NSAIDs were 0.68 (0.57–0.81), 0.80 (0.67–0.95), and 0.65 (0.50–0.85), respectively.

Aspirin may prevent heart disease (Hennekens and Schneider, 2008) and colon cancer (Dubé *et al*, 2007), but the US Preventive Services Task Force does not endorse an aspirin regimen for primary chemoprevention of colon cancer (US Preventive Services Task Force, 2007). Daily aspirin use carries the risk of gastrointestinal bleeding and haemorrhagic stroke and the expected benefits do not outweigh the risks, at least in individuals at average risk for colorectal cancer. If additional chemopreventive benefits at sites other than the colon could be included in the risk benefit analysis, this calculation may change.

Worldwide, gastric cancer remains the second leading cause of death due to cancer, with over 900 000 incident cases and about 700 000 deaths (Parkin *et al*, 2002). Although gastric cancer incidence rates are decreasing in the United States, about 21 000 incident cases occur each year (Ries and Melbert, 2007). In contrast, oesophageal adenocarcinoma rates have increased dramatically over the last 30 years in many Western countries, and there are about 9000 incident cases in the United States each year (Ries and Melbert, 2007). Both stomach and oesophageal cancers have high fatality rates, only 24 and 16% 5-year survival respectively, so preventive strategies are particularly important for these cancers. Earlier studies suggest that the incidence of adenocarcinomas of the oesophagus and stomach may be reduced by the use of aspirin or other non-steroidal anti-inflammatory drugs (NSAIDs) (Corley *et al*, 2003; Gonzalez-Perez *et al*, 2003; Wang *et al*, 2003), but few studies have been prospective, used data collected directly from subjects, and controlled for the many potential confounders.

We aimed to examine the association between aspirin and non-aspirin NSAID use and risk of oesophageal, gastric cardia, and gastric non-cardia adenocarcinoma in the NIH-AARP Diet and Health Study cohort, a large prospective study conducted in the United States.

## Methods

The NIH-AARP Diet and Health Study is a large prospective cohort study designed to investigate the association between diet and other factors and risk of cancer and has been described in detail previously (Schatzkin *et al*, 2001). Between 1995 and 1996, a questionnaire was mailed to 3.5 million AARP members (aged 50–71 years) in eight US states (California, Florida, Georgia, Louisiana, Michigan, New Jersey, North Carolina, and Pennsylvania). In total, 617 119 individuals returned the questionnaire. A second mailed questionnaire (1996–1997) collected additional information including NSAID use and 334 908 individuals were available for analysis. We excluded subjects for whom either the baseline (*N*=6959) or the follow-up questionnaire (*N*=3424) was completed by proxies, those with prevalent cancer at the second questionnaire baseline (*N*=4543), those with incomplete information about NSAID use (*N*=6353), those with total energy intake more than twice the interquartile range from the median (*N*=2506), and those who exited on the first day of follow-up (*N*=8). The resulting cohort included 311 115 participants including 180 337 men and 130 778 women. The NIH-AARP Diet and Health Study was approved by the Special Studies Institutional Review Board of the US National Cancer Institute.

As described earlier (Michaud *et al*, 2005), addresses for members of the NIH-AARP cohort were updated annually by matching the cohort database to that of the National Change of Address database maintained by the US Postal Service. We ascertained vital status by annual linkage of the cohort to the Social Security Administration Death Master File, cancer registry linkage, questionnaire responses, and responses to other mailings. Incident cases of cancer through the year 2003 were identified by probabilistic linkage between the NIH-AARP cohort membership and the cancer registry databases of the states of residence at the time of mailing the questionnaire with the addition of Arizona, Nevada, and Texas, each of which has been certified by the North American Association of Central Cancer Registries for meeting the highest standards of data quality. For matching purposes, first and last name, address history, gender, date of birth, and Social Security number (available for 85% of our participants) were used. We estimate the sensitivity of case identification to be about 90%. Cancer sites were identified by anatomic site and histologic code as detailed earlier (Freedman *et al*, 2007), using the International Classification of Disease for Oncology, third edition. We classified tumours with site codes C15.0–C15.9 as oesophageal cancers, site code C16.0 as gastric cardia tumours, and site codes C16.1–C16.9 as non-cardia tumours. All included cancers were classified as adenocarcinomas.

Our questionnaire assessed aspirin (generic aspirin and trade names) use and non-aspirin NSAID use separately and the latter named 19 non-aspirin NSAIDs (e.g., ibuprofen, sulindac etc., using both generic and trade names) and specifically excluded Tylenol, acetaminophen, and other pain relievers. Both questions asked about any use in the past 12 months and asked users to mark how frequently they took them: less than two times per month, two to three times per month, one to two times per week, three to four times per week, five to six times per week, one time per day, or two or more times per day. Owing to small numbers in some of the categories, we collapsed these into monthly, weekly, or daily use.

The baseline questionnaire contained questions about demographic information, cigarette use, alcohol consumption, education, and a food frequency questionnaire of 124 items. Potential confounders were categorised as described earlier (Abnet *et al*, 2008).

### Statistics

We computed two-sided tests and considered *P*-values <0.05 or estimates with confidence intervals (CIs) that excluded 1.0 as statistically significant. To assess the potential for confounding, we tabulated and compared known or potential risk factors by NSAID use status. Hazard ratios (HRs) and 95% CIs were calculated using Cox proportional hazards regression with follow-up time as the underlying time metric using SAS 9.1 (Cary, NC, USA). All presented estimates come from models adjusted for age at cohort entry, sex, cigarette smoking status and intensity, alcohol use, education, fruit intake, vegetable intake, BMI, total energy intake, and both vigorous physical activity and usual physical activity throughout the day. A small number of subjects were missing values for some adjusting covariates and these subjects were retained using dummy variables in the models. We modelled the HR (95% CI) for any use of aspirin and of non-aspirin NSAIDs in a single model (i.e., mutually adjusted models) and for frequency of use with adjustment for the other category of NSAIDs. We tested the proportional hazards assumption using cross-product terms for interaction between follow-up time and any use of NSAIDs and found no significant deviations from proportionality. Furthermore, we dropped 1, 2, or 3 years of initial follow-up and refitted the models to assess lag effects and found none. Age-standardised incidence rates were calculated as in Breslow and Day (1987) with 5-year age bands and age- and sex-specific rates standardised to the entire NIH-AARP Diet and Health Study population.

### Meta-analysis

To conduct the meta-analysis, we searched PubMed on 20 November 2008, with the following terms: (aspirin OR nsaid OR nsaids OR non-steroidal) AND (gastric cancer OR oesophageal cancer) AND (case-control OR cohort OR epidemiologic). We reviewed the 128 retrieved abstracts to find relevant papers, reading those in full in which the abstracts were not entirely informative. We also reviewed earlier meta-analyses on aspirin and NSAIDS in relation to oesophageal and gastric cancers (Corley *et al*, 2003; Gonzalez-Perez *et al*, 2003; Wang *et al*, 2003) and other review articles (Baron, 2003; Bosetti *et al*, 2003). We limited our analysis to papers reporting case–control or cohort studies of the association between the use of either aspirin or non-aspirin NSAIDS and the risk of oesophageal or gastric adenocarcinomas. We included the following studies for the oesophagus (Farrow *et al*, 1998; Cheng *et al*, 2000; Lindblad *et al*, 2005; Anderson *et al*, 2006; Jayaprakash *et al*, 2006; Ranka *et al*, 2006; Fortuny *et al*, 2007; Duan *et al*, 2008; Sadeghi *et al*, 2008), cardia (Farrow *et al*, 1998; Zaridze *et al*, 1999; Akre *et al*, 2001; Fortuny *et al*, 2007; Duan *et al*, 2008; Sadeghi *et al*, 2008), non-cardia (Farrow *et al*, 1998; Zaridze *et al*, 1999; Akre *et al*, 2001; Fortuny *et al*, 2007; Duan *et al*, 2008), and gastric NOS (Thun *et al*, 1993; Schreinemachers and Everson, 1994; Coogan *et al*, 2000; Langman *et al*, 2000; Akre *et al*, 2001; Sorensen *et al*, 2003; Ratnasinghe *et al*, 2004; Lindblad *et al*, 2005) and excluded a few studies from certain sections that did not specify the agent (Garidou *et al*, 1996; Suleiman *et al*, 2000) or the histology of the oesophageal tumours (Funkhouser and Sharp, 1995; Langman *et al*, 2000). Two other studies, one prospective and one retrospective, reported on the association between aspirin and oesophageal adenocarcinoma, but included only subjects with Barrett's oesophagus in the reference group (Tsibouris *et al*, 2004; Vaughan *et al*, 2005) and these papers are discussed separately. From each selected paper, the effect measures (odds ratio (OR) or HR) and 95% CIs were tabulated by two investigators. In each case, we selected the most expansive measure of NSAID use and the maximally adjusted model that did not include adjustment for reflux symptoms (where possible). In some cases, we collapsed multiple exposure groups into a single measure of association using fixed effects models, and these are indicated by asterisks in the figure. We used Stata/SE version 8.0 (Stat Corp., College Station, TX, USA) and the meta and metabias commands to complete the analyses. We report the results from random effects models, but the results for fixed effects are similar in each case. Plots were created using SigmaPlot 8.0 (Systat Corp., Chicago, IL, USA).

## Results

In the 12 months before baseline, 73% of the cohort had used aspirin and 56% had used non-aspirin NSAIDs (Table 1), 25% reported daily aspirin use and 10% reported daily non-aspirin NSAID use. Aspirin users and non-aspirin NSAID users appeared similar to each other and to the cohort as a whole in their age, smoking histories, alcohol-drinking habits, education, diet, BMI, and amount of physical activity.

The cohort members had an average of 6.7 years of follow-up and we collected 2 078 248 person years of follow-up in total. The HRs and 95% CIs for both any use and the frequency of aspirin or non-aspirin NSAID use are given in Table 2. Models adjusted for only age and sex produced similar results to these fully adjusted models. For gastric non-cardia cancer, we found a strong dose-dependent protective association for aspirin. Any aspirin use had an HR (95% CI) of 0.64 (0.47–0.86). The HRs decreased from 0.74 for monthly use to 0.57 for weekly or daily use and the test for trend across categories was significant (*P*=0.0032). For non-aspirin NSAIDs, any reported use showed a significantly decreased risk of non-cardia gastric cancer, 0.68 (0.51–0.92). But there was no clear trend; the HRs were 0.71, 0.48, and 0.82, for monthly, weekly, and daily use, respectively, and the test for trend across categories was borderline non-significant (*P*=0.050). We saw no significant associations between any use of aspirin (1.00, 0.73–1.37) or non-aspirin NSAIDs (0.90, 0.69–1.17) and the risk of oesophageal adenocarcinoma.

We combined aspirin and non-aspirin NSAIDs into a single category and found statistically significantly reduced risk of non-cardia gastric cancer, 0.64 (0.44–0.91), compared with never using either agent, but no significant associations with risk of cancer at the other two sites. As adenocarcinomas of the oesophagus and cardia can be difficult to separate, we combined these two sites into a single outcome, but still found no significant associations with either aspirin or non-aspirin NSAID use. We adjusted for and also stratified by reported use of antacids, a proxy marker for reflux disease or heartburn, and found no differences compared with the presented results (data not shown). We tested for and found no evidence of a significant interaction between sex and NSAID use. Finally, we deleted the first 1, 2, and 3 years of follow-up and found similar results to those for the full follow-up period.

We calculated age-standardised incidence rates for non-cardia gastric cancer in aspirin users and non-users. The rates (95% CI) per 100 000 person years dropped from 11.0 (8.4–13.6) in non-aspirin users to 7.0 (5.7–8.3) in users. We also calculated rates in men and women separately because of the underlying difference in risk for this cancer. The number of non-cardia gastric cases in women in our cohort was small (*N*=53), and among aspirin non-users and users we found rates of 6.4 (3.7–9.1) and 5.1 (3.3–6.9), respectively. In men (*N*=129), we found rates of 16.4 (11.6–21.1) and 8.2 (6.4–9.9), respectively.

**Figure 1:**
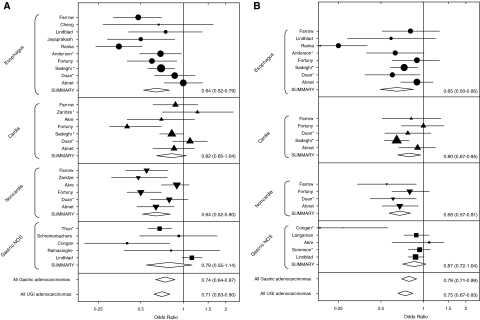
Forest plots for the association between any aspirin (**A**) or non-aspirin NSAID (**B**) use and risk of oesophageal, gastric cardia, or gastric non-cardia cancer. Summary estimates and study weights (proportional to symbol size) come from random effects models. Studies are listed by the last name of the first author and Abnet refers to this study. We used the broadest measure of NSAID exposure and multivariable-adjusted estimates whenever possible. To make the exposure measures more comparable, we generated new combined estimates of effect when the published estimates were stratified on dose or duration, and this is indicated by an asterisk after the first author's name. Gastric NOS means that the location of the tumours within the stomach was not specified. The summary estimate for all studies included in this figure was 0.72 (0.67–0.79).

**Figure 2:**
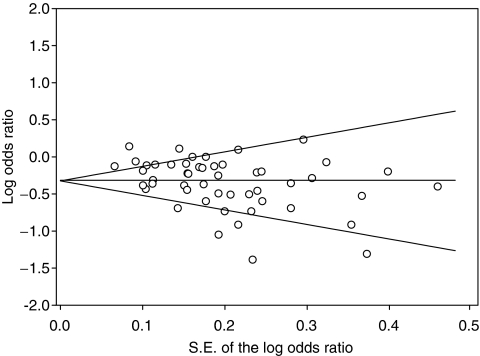
Begg funnel plot with pseudo 95% confidence intervals for all estimates included in the meta-analysis of NSAID use and oesophageal or stomach adenocarcinoma. Both the Begg test (*P*=0.010) and the Egger test (*P*=0.001) suggested publication bias, but, dropping the studies with a standard error greater than 0.2 (*N*=20) or a log OR less than −0.50 (*N*=14) left the association essentially unchanged. Therefore, publication bias probably had little effect on the summary estimates.

To put our results in a larger perspective, we completed a meta-analysis of 49 risk estimates from 17 published studies reporting the association between either aspirin (Figure 1A) or non-aspirin NSAID (Figure 1B) use and the risk of oesophageal or gastric adenocarcinoma. In the meta-analysis, aspirin use was inversely associated with both gastric and oesophageal adenocarcinomas, with summary ORs (95% CI) for non-cardia, cardia, and oesophageal adenocarcinomas of 0.64 (0.52–0.80), 0.82 (0.65–1.04), and 0.64 (0.52–0.79), respectively. The corresponding numbers for other NSAIDs were 0.68 (0.57–0.81), 0.80 (0.67–0.95), and 0.65 (0.50–0.85), respectively. Figure 2 shows a Begg funnel plot for detecting publication bias in all the NSAID and upper GI adenocarcinoma literature combined. The summary OR (95% CI) for all estimates included in Figure 1A and B was 0.72 (0.67–0.79). Using either the Begg test (*P*=0.019) or the Egger test (*P*=0.002), we found evidence of publication bias and the figure suggests that the current literature may overestimate the beneficial effects of NSAIDs. But, when we excluded the smaller studies (those with standard error greater than 0.2; *N*=20) or those that showed strong protection (log OR less than −0.50; *N*=14), we found that the association was essentially unchanged.

## Discussion

We found that reported use of aspirin or non-aspirin NSAIDs was associated with a significant 36% reduction in the risk of non-cardia gastric cancer consistent with the earlier studies. For ever use of aspirin in the previous 12 months, age-adjusted rates of gastric non-cardia cancer dropped from 11.0 in non-users to 7.0/100 000 person years in users. Although we did not find a significant association between use of aspirin or other NSAIDs with cardia cancer, the point estimate in our study was very close to the summary estimate from the meta-analysis, which showed a protective effect. Unlike most earlier observational studies, we found no such association with oesophageal adenocarcinoma.

Our finding in the NIH-AARP cohort study that aspirin or other NSAIDs had a protective association with gastric non-cardia adenocarcinoma is consistent with the literature published earlier, which is summarised in Figure 1. It appears that aspirin and non-aspirin NSAIDs have similar effects, which may have implications for cancer prevention. Eradication of *Helicobacter pylori*, the strongest risk factor for gastric non-cardia adenocarcinoma, may reduce its incidence (Talley, 2008). However, recent studies suggest that *H. pylori* may have health benefits, such as preventing asthma (Blaser *et al*, 2008) or oesophageal adenocarcinoma (Islami and Kamangar, 2008). Beyond the direct benefits and risks of eradication to the individual, the methods and consequences of attempted widespread eradication, such as increasing antibiotic resistance, must also be considered (Graham and Shiotani, 2008). A single trial has tested the effect of the NSAID rofecoxib on subjects with gastric pre-neoplastic lesions over 24 months and found no evidence of benefit, but this study was small and did not use cancer as an end point (Leung *et al*, 2006). The remarkably consistent observational results showing that NSAID use is associated with a reduced risk of gastric cancer may warrant a randomised trial in a suitable population at high risk for the disease in which side effects can be monitored closely.

The epidemiology of gastric cardia tumours in the United States is similar to that of oesophageal adenocarcinoma. The incidence of this tumour may have increased in recent years, but this change may have occurred because of changing patterns of diagnosis or because of the difficulty of separating adenocarcinomas in the gastric cardia from those in the oesophagus (Kubo and Corley, 2002). We found no significant association between use of either aspirin or non-aspirin NSAIDs and risk of gastric cardia adenocarcinoma, but our point estimates are similar to the summary estimates from our meta-analysis, which suggests a significant protective effect.

We found no evidence that ever or daily aspirin use lowered the risk of oesophageal adenocarcinoma, for which, as shown in Figure 1, our results are discordant with many earlier studies. Most of these showed some evidence, albeit not always significantly, that use of aspirin or non-aspirin NSAIDs was associated with reduced risk.

The reasons for these differences are not clear. Most earlier studies had retrospective designs and may be prone to reverse causality for NSAIDs, as subjects with reflux symptoms, and therefore at higher risk of oesophageal adenocarcinoma, may avoid using NSAIDs producing the appearance of protection among users. But at least one earlier prospective study found that NSAID use reduced risk of progression to oesophageal adenocarcinoma among subjects with Barrett's oesophagus (Vaughan *et al*, 2005). Recently, in the same cohort, the association is found strongest among subjects with multiple molecular abnormalities that confer the greatest risk of progressing to cancer, but absent in those at the lowest risk (Galipeau *et al*, 2007). One study using subjects with Barrett's oesophagus as controls found that oesophageal adenocarcinoma cases and subjects with Barrett's used aspirin and NSAIDs at similar rates, but this differed with long-term reflux symptoms (Tsibouris *et al*, 2004). One small, short-term trial tested the effect of twice-daily treatment with 200 mg of celecoxib for 48 weeks on the proportion of dysplastic biopsies in subjects with Barrett's oesophagus and did not find any benefit (Heath *et al*, 2007). A large trial of proton pump inhibitors with or without aspirin for the chemoprevention of oesophageal adenocarcinoma in men with Barrett's oesophagus is underway (Jankowski and Moayyedi, 2004).

Our study has several strengths, being based on a large prospective cohort that provided adequate power and minimised recall bias, which may alter the associations found in case–control studies. We used subject-completed questionnaires that captured both over-the-counter and pharmacy-provided NSAIDs and information on many potentially confounding exposures, many of which (e.g., age, tobacco smoking, and physical activity) were similar among NSAID users and non-users. On the other hand, we captured only NSAID use over the previous 12 months and did not collect the duration of use, which could have caused misclassification of subjects who, for example, recently ceased NSAID use due to upper gastrointestinal symptoms. But, we did adjust for and stratify by antacid use without change in our risk estimates. We could not assess infection with *H. pylori* and infected subjects may have different patterns of NSAID use, which would lead to different confounding effects in the stomach and oesophagus. Finally, this study, being observational, is susceptible to confounding by other unmeasured or poorly measured confounders, supporting the need for randomised controlled trials.

In this large prospective cohort study, we found further evidence that regular use of aspirin or non-aspirin NSAIDs may reduce the risk of non-cardia gastric cancer; in contrast, this was not associated with reduced risk of oesophageal adenocarcinoma, thereby differing from most earlier studies.

## Figures and Tables

**Table 1:** Distribution of covariates in NSAID users and non-users in NIH-AARP Diet and Health Study

**Variable**	**Cohort**	**Aspirin users in the past 12 months**	**Non-aspirin NSAID users in the past 12 months**
Number	311 115 (100%)	227 198 (73%)	175 591 (56%)
Age, years, mean (s.d.)	62.3 (5.3)	62.3 (5.3)	61.7 (5.4)
Sex, male, *N* (%)	180 337 (58%)	141 387 (62%)	97 001 (55%)
			
*Tobacco smoking*			
Never	111 128 (37%)	79 079 (36%)	61 947 (36%)
Former<20 cigarettes per day, *N* (%)	84 125 (28%)	61 840 (28%)	48 356 (28%)
Former⩾20 cigarettes per day, *N* (%)	66 306 (22%)	50 395 (23%)	37 966 (22%)
Current<20 cigarettes per day, *N* (%)	25 506 (8%)	18 088 (8%)	14 150 (8%)
Current⩾20 cigarettes per day, *N* (%)	13 924 (5%)	10 323 (5%)	7505 (4%)
			
*Education*			
High school or less, *N* (%)	72 276 (24%)	49 679 (22%)	38 088 (22%)
Post-high school training, *N* (%)	103 305 (34%)	75 067 (34%)	58 775 (34%)
College graduate, *N* (%)	61 520 (20%)	46 410 (21%)	35 516 (21%)
Post-graduate education, *N* (%)	66 493 (22%)	50 702 (23%)	39 087 (23%)
			
Alcohol, drinks per day, mean (s.d.)	0.9 (2.3)	1.0 (2.3)	0.9 (2.1)
Fruit intake, servings per day, mean (s.d.)	3.0 (2.4)	2.9 (2.3)	2.9 (2.3)
Vegetable intake, servings per day, mean (s.d.)	3.9 (2.4)	3.9 (2.4)	3.9 (2.4)
Body mass index, kg m^−2^, mean (s.d.)	26.9 (5.0)	26.9 (4.8)	27.2 (5.1)
			
*Vigorous physical activity*			
Never, *N* (%)	12 309 (4%)	7619 (3%)	6154 (4%)
Rarely, *N* (%)	40 323 (13%)	27 952 (12%)	22 580 (13%)
1–3 times per month, *N* (%)	41 327 (13%)	30 307 (13%)	24 164 (14%)
1–2 times per week, *N* (%)	66 624 (22%)	49 501 (22%)	38 486 (22%)
3–4 times per week, *N* (%)	85 859 (28%)	64 141 (28%)	49 257 (28%)
⩾5 times per week, *N* (%)	62 226 (20%)	46 028 (20%)	33 710 (20%)
			
*Activity throughout the day*			
Sit during day, not much walking, *N* (%)	23 614 (8%)	17 063 (8%)	13 887 (8%)
Sit much of the day, walk a fair amount, *N* (%)	74 037 (33%)	74 037 (33%)	58 204 (34%)
Stand/walk a lot, no lifting, *N* (%)	118 404 (39%)	86 292 (39%)	65 899 (38%)
Lift carry light loads, *N* (%)	54 906 (18%)	40 220 (18%)	30 199 (18%)
Heavy work, *N* (%)	7874 (3%)	5670 (3%)	4331 (3%)

Abbreviation: NSAID=non-steroidal anti-inflammatory drug.

**Table 2:** Hazard ratios (95% CI)^a^ for the association between NSAID use and the risk of cancer in the NIH-AARP Diet and Health Study

	**Any aspirin use in the past 12 months**				**Frequency of aspirin use in the past 12 months**			
	**No**	**Yes**		**None**	**Monthly**	**Weekly**	**Daily**	
**Cohort, *N* (%)**	83 917 (27%)	227 198 (73%)		83 917 (27%)	96 863 (31%)	52 096 (17%)	78 239 (25%)	
			**HR (95% CI)**	**Reference**	**HR (95% CI)**	**HR (95% CI)**	**HR (95% CI)**	***P*_trend_ b**
Oesophageal adenocarcinoma, *N* (%)	52 (23%)	176 (77%)	1.00 (0.73–1.37)	1.0	0.95 (0.65–1.37)	0.91 (0.59–1.40)	1.11 (0.78–1.57)	0.52
Gastric cardia adenocarcinoma, *N* (%)	48 (27%)	130 (73%)	0.86 (0.61–1.20)	1.0	0.80 (0.53–1.20)	0.71 (0.43–1.18)	0.99 (0.67–1.45)	0.96
Gastric non-cardia adenocarcinoma, *N* (%)	67 (37%)	115 (63%)	0.64 (0.47–0.86)	1.0	0.74 (0.51–1.07)	0.57 (0.35–0.92)	0.57 (0.39–0.85)	0.0032
								

	**Any non-aspirin NSAID use in the past 12 months**				**Frequency of non-aspirin NSAID use in the past 12 months**			
	**No**	**Yes**		**None**	**Monthly**	**Weekly**	**Daily**	
**Cohort, *N* (%)**	135 524 (44%)	175 591 (56%)		135 524 (44%)	102 165 (33%)	41 568 (13%)	31 858 (10%)	
			**HR (95% CI)**	**Reference**	**HR (95% CI)**	**HR (95% CI)**	**HR (95% CI)**	***P*_trend_**
Oesophageal adenocarcinoma, *N* (%)	113 (50%)	115 (50%)	0.90 (0.69–1.17)	1.0	0.87 (0.64–1.19)	0.99 (0.65–1.51)	0.89 (0.55–1.43)	0.62
Gastric cardia adenocarcinoma, *N* (%)	88 (49%)	90 (51%)	0.91 (0.67–1.22)	1.0	0.83 (0.59–1.19)	1.13 (0.72–1.78)	0.87 (0.51–1.48)	0.80
Gastric non-cardia adenocarcinoma, *N* (%)	106 (58%)	76 (42%)	0.68 (0.51–0.92)	1.0	0.71 (0.50–1.02)	0.48 (0.26–0.87)	0.82 (0.50–1.34)	0.050

Abbreviations: BMI=body mass index; CI=confidence interval; HR=hazard ratio; NSAID=non-steroidal anti-inflammatory drug.

a HRs and 95% CIs come from models adjusted for age at cohort entry, sex, cigarette smoking status, alcohol, education, fruit intake, vegetable intake, BMI, total energy intake, and both vigorous physical activity and usual physical activity throughout the day. Estimates for any aspirin and any non-aspirin NSAID are from a single model (i.e., mutually adjusted) and those for frequency of use were adjusted for any use of the other class of NSAID.

b Trend tests used the category of intake as an ordinal variable (0–3).
